# Encyclopaedia of eukaryotic DNA methylation: from patterns to mechanisms and functions

**DOI:** 10.1042/BST20210725

**Published:** 2022-05-06

**Authors:** Peter Sarkies

**Affiliations:** 1 Department of Biochemistry, University of Oxford, Oxford, U.K.; 2 MRC London Institute of Molecular Biology, London, U.K.; 3 Institute of Clinical Sciences, Imperial College London, London, U.K.

**Keywords:** comparative genomics, epigenetics, evolutionary biology, methylation, transposons

## Abstract

DNA methylation is an epigenetic modification with a very long evolutionary history. However, DNA methylation evolves surprisingly rapidly across eukaryotes. The genome-wide distribution of methylation diversifies rapidly in different lineages, and DNA methylation is lost altogether surprisingly frequently. The growing availability of genomic and epigenomic sequencing across organisms highlights this diversity but also illuminates potential factors that could explain why both the DNA methylation machinery and its genome-wide distribution evolve so rapidly. Key to this are new discoveries about the fitness costs associated with DNA methylation, and new theories about how the fundamental biochemical mechanisms of DNA methylation introduction and maintenance could explain how new genome-wide patterns of methylation evolve.

## Introduction

Bases in DNA across all domains of life are often modified by a variety of chemical moieties. Probably the most widespread and well-characterised modification is methylation. In eukaryotes, methylation predominantly is found at the five position of cytosine bases, but other forms of methylation, including different positions on cytosine and on adenine have been documented.

The enzymatic properties of DNA methyltransferases have been extensively reviewed elsewhere [1]. Here I will discuss the extraordinary variation in the machinery and genome-wide patterns of DNA methylation across eukaryotic organisms. I will attempt to show how we are starting to move beyond a characterisation of the evolution of DNA methylation levels and patterns towards an understanding of the factors shaping this diversity across species. Remarkably, rapid evolution of DNA methylation patterns occurs not only across evolution, but also within human cancers, where the genome-wide distribution of DNA methylation is often vastly different from the cell of origin. Understanding the factors that drive DNA methylation evolution across species may give insights into why DNA methylation evolves so fast in cancer, and the potential functional consequences.

### Types of cytosine-5 methylation (5mC)

The most widespread and abundant form of methylation across eukaryotic species is cytosine-5 methylation at CG sequences (also referred to as CpG methylation). CG methylation is a paradigm to understand how methylation of DNA can be heritable through cell division without requirement on the initial trigger [2–4]. CG methylation is introduced onto both strands of unmodified CG sequences by enzymes known as *de novo* methyltransferases. In mammals, these are known as DNMT3A and DNMT3B. Homologues of DNMT3 are found in early land plants; the enzyme DRM is also likely derived from DNMT3 [1,4]. Every time the cell divides new, unmodified CG bases are introduced opposite methylated CG. However, another methyltransferase enzyme, DNMT1 in mammals, shows high affinity for the ‘hemi-methylated' intermediate where one strand retains methylation [3,4]. This mechanism of CG methylation maintenance is conserved in plants, where the homologous enzyme is known as MET1 or DMT1, and in fungi [5], indicating that it must have evolved very early in eukaryotes [6].

DNMT5 is an additional DNMT found in some fungi and algae. DNMT5 is a CG methyltransferase [7]. In *Cryptococcus neoformans*, DNMT5 has maintenance activity and has no *de novo* activity [8]; however its substrate preferences in other organisms are undetermined. Additional methyltransferases, found in some fungi and protists are DNMT4 and DNMT6 [5]. Their functions are not yet clear but at least DNMT6 does not seem to be a DNA methyltransferase [9].

5mC can occur in other sequence contexts besides CG. In the model land plant *Arabidopsis*, ∼7% of cytosines in the CHG context and 2% in the CHH context are methylated [10]. DMT2 alongside two dedicated enzymes, CMT2 and CMT3 introduce CHH and CHG methylation [11,12]. Despite no maintenance mechanisms CHG and CHH methylation can persist through cell division because they take part in a feedback loop involving small RNAs and chromatin modifications [4,13].

In mammals, particularly in brain, non-CG methylation also occurs [14,15]. This is conserved across vertebrates; however, the levels of this methylation are much lower than CG methylation, and it is largely dependent on DNMT3A rather than enzymes specific for non-CG contexts [16]. It is therefore possible that non-CG methylation reflects off-target activity of CG DNMTs. As cells in the brain do not divide, this would not be removed through dilution during cell division, hence its particular accumulation in brain tissue. This possibility can be countered by the rather high abundance of non-CG methylation. Moreover, specific proteins, notably the Rett syndrome protein MeCP2, recognise CAA and CAT methylation [17]. Thus it seems plausible that non-CG methylation is functionally relevant but the exact role remains to be determined.

### Factors influencing the levels of cytosine DNA methylation across eukaryotes

DNMT1 and DNMT3 have been lost from many distinct eukaryotic lineages including several model organisms including *C. elegans*, *Drosophila*, *S. cerevisiae* and *S. pombe* [5,6]. Many of these species still retain TRDMT1, a homologue of DNMT1 and DNMT3, originally known as was DNMT2 [5,6]. However, this enzyme does not act on DNA efficiently *in vitro* and instead methylates tRNA, with Aspartate tRNA a prominent substrate [18]. Some evidence from mass spectrometry and bisulfite sequencing suggested that TRDMT1 might result in very low levels of DNA methylation (<1% of cytosines) [19–21]. However, these findings can be questioned. Bisulfite sequencing relies on conversion of unmethylated cytosine into uracil by sodium bisulfite treatment and there is often a substantial non-conversion rate of between 0.1 and 1% of cytosines [22]. Mass spectrometry is potentially able to rigorously assess low levels of DNA methylation with very high sensitivity and accuracy but contamination from reagents used to prepare samples is common [23]. Recent studies suggest that DNA methylation is undetectable in species with only TRDMT1 and mammalian cells without DNMT1, 3A and 3B but retaining TRDMT1 [24].

Even if there is some low-level cytosine methylation provided by TRDMT1, complete loss of DNA methylation has occurred frequently across evolution- *C. elegans* and *S. cerevisiae*, for example, lack DNMT1, 3 and TRDMT1 [5]. This might hint at the possibility of an evolutionary cost associated with DNA methylation.

One possible cost of DNA methylation is the mutagenic nature of methylated cytosine. CG sites in vertebrates are mostly methylated. In almost all vertebrate species examined they are less common than would be expected given the occurrence of C and G across the genome [25]. In insect species where a subset of genes are methylated, methylated genes have strongly depleted CG frequency compared with unmethylated genes [26]. Consistently, analyses of human somatic mutations in cancer and normal tissue demonstrate that the highest rate of mutation is C-T mutations at methylated CG sites [27]. The source of the excess mutagenicity of methylated cytosine is most likely to be that methylated cytosine deaminates to thymine rather than uracil [28–30]. High mutation rate is disadvantageous [31], thus may promote evolutionary loss of DNA methylation [32].

New insights into costs associated with DNA methylation came from the discovery that DNMT1 and DNMT3 co-evolve with a DNA repair enzyme, ALKB2, across eukaryotes. ALKB2 and, in mammals, its paralogue ALKB3, has evolved to repair 3mC in DNA [33]. 3mC is a highly toxic form of DNA damage because of its ability to interfere with base pairing; it leads to a replication fork stall and potentially double-strand breaks [34,35]. The co-evolution was explained by the finding that DNMTs directly introduce 3mC as an off-target effect both *in vitro* and in mammalian cells [36]. Modelling of the catalytic site of DNMT3A suggests that this is an inevitable property of the enzymatic mechanism [37]. Lower levels of 3mC might be advantageous to fast-dividing cells as there would be insufficient time to repair 3mC, even with ALKB2 present, before replication happens. This might lead to a drive to lose DNMTs in organisms with fast early embryonic divisions such as nematodes and many insects.

When DNA methylation is present, its levels vary enormously across different organisms. A striking example of this is the arthropod phylum. The first arthropods discovered to have DNA methylation were honey bees, where the overall percentage of methylation is very low at ∼1% of CG sites [26,38]. This led to a view that arthropod methylation was ‘sparse' with most genes totally devoid of methylation with a few highly methylated genes and little methylation in intergenic regions or repetitive elements such as transposons [39–42]. However, further work including studies of individual species [43–46], a study across insects [47] and an attempt to systematically examine many branches of arthropods [48], has shown that there is a huge range of methylation within arthropods. The centipede *S. maritima* possesses much higher levels of DNA methylation at ∼30% of CGs [46,48], whilst methylation occurs only at ∼0.5% of CG sites in the burying beetle *Nicrophorus vespilloides* [49].

One factor that is associated with different levels of DNA methylation across arthropods is the alkylation repair enzyme ALKB2. In most organisms, the presence or absence of DNMT1 and 3 correlates with the presence or absence of ALKB2 [36]. In arthropods, the level of methylation correlates with the presence of ALKB2 [48]. This indicates that species that methylate a very small proportion of their cytosines correspondingly produce a smaller level of 3mC and therefore do not require ALKB2 activity [48]. A similar relationship, although not as strong, exists in fungi [50]. It will be interesting to determine whether this relationship also affects the expression of ALKB2 in different species depending on different methylation levels.

Global analyses of methylation in whole animals or selected tissues may mask more subtle differences in DNA methylation levels between tissues or during development. Development in mammals shows dynamic changes in DNA methylation. During embryonic development there are two waves of demethylation where the average methylation, which is ∼70% of CGs in adult somatic tissues, drops from ∼10% in the zygote to 1% before rising again [51]. The dramatic remodelling of DNA methylation in mammals is not conserved across vertebrates [52], and therefore probably evolved to facilitate imprinting, a process where methylation levels and gene expression of ∼250 genes varies depending on whether they are on the maternal or paternal chromosome [53].

## Variety in genome-wide methylation patterns

In addition to the variability in methylation levels across species, the sites which are methylated within the genome vary in their genomic location. There are a few recognised archetypes, which are summarised in Table 1. However, the increasing number of species with methylation patterns that have been analysed by genome-wide bisulfite sequencing throws some doubt on the extent to which all of these archetypes are ancestral properties of eukaryotic DNA methylation.

**Table 1:** Summary of key forms of DNA methylation across eukaryotes

Type	Presumed function	Phylogenetic distribution
TE methylation	Silencing of mobile elements to ensure genome stability	Vertebrates; Plants; some Arthropods; Nematodes; Fungi; Sponges
Gene body methylation	Unclear- proposals include suppressing intergenic transcription and that it has no explicit function in gene expression	Widespread; exceptions are fungi, nematodes, basal plants.
Promoter methylation	Gene expression control	Vertebrates; Centipedes; Mealybugs; Sponges; Flowering Plants
Repeat-induced point mutation (RIP)	Destruction of mobile elements to ensure genome stability	Fungi-mostly Ascomycota
Periodic methylation by DNMT5	Genome compaction?	Algae

### DNA methylation in deuterostomes

Extensive studies of DNA methylation in mammalian cells demonstrated extremely high levels of methylation at ∼80% of cytosines in the CG context are methylated [54]. All the categories of DNA methylation present in Table 1 are found in mammals. DNA methylation of transposable elements (TEs) is required for robust silencing, as many TEs become desilenced when DNMTs are mutated [55]. Promoter methylation in mammals is associated with silencing of genes, often in a developmental context or at imprinted genes [56]. Failure of DNA methylation in development is lethal [57,58]. Further on in development genes are shut down in specific cells as part of differentiation [56]. It is important to note that DNA methylation acts in concert with many other epigenetic factors such as histone modifications, non-coding RNAs and nuclear localisation to maintain silencing of developmentally inactivated genes; positive feedback between all these epigenetic factors ensures robust silencing [59].

The role of methylation within genes, known as ‘gene-body' methylation, is more nuanced. Gene-body methylation is generally weakly associated with transcriptional activity, but this is not the case for all tissues [54,60]. In mammals DNMT3B carries a PWWP domain that binds to H3K36me3 modified nucleosomes, a modification associated with transcription, thus may be responsible for methylation being slightly higher at expressed genes [61]. Gene body methylation is hypothesised to play a role in preventing spurious transcription initiation from within the gene [61,62].

How ancient is the mammalian pattern of DNA methylation? Methylation within genes, transposons and promoters is found in chicken [63], zebrafish [64] and Xenopus [52]. However, the early-branching chordate *Branchiostoma lanceolatum* (Amphioxus) has methylation predominantly located within genes, with no evidence of extensive promoter methylation or TE methylation [65]. Similar patterns were observed in *Ciona intestinalis* [39]. Interestingly the early-branching chordate *Oikilopleura* has lost DNMT1 and DNMT3 [66]; despite this a study reported moderate levels of DNA methylation genome-wide [67]. This work was carried out using an immunoprecipitation approach and should probably be re-evaluated using bisulfite sequencing. In both Amphioxus and *Ciona*, there is a correlation between gene activity and DNA methylation [39,65].

Further back in deuterostome evolution, the sea urchin *Strongylocentrotus purpuratus* displays a similar pattern of DNA methylation to early-branching chordates. DNA methylation is found at a subset of expressed genes but apparently absent from TEs and promoters [68]. On this basis a reasonable conclusion would be that the genome-wide methylation pattern seen in vertebrates was not present in the ancestral deuterostomes. However, studying further species by bisulfite sequencing would be important to confirm this hypothesis. At present it is difficult to speculate about what changes in ecology or genome structure could have led to this change in DNA methylation [42,69]. Notably, TEs are abundant in the amphioxus genome [65], thus it is unlikely that the high repeat content that is a feature of vertebrate genomes is sufficient to explain the evolution of high genome-wide methylation levels.

### DNA methylation in nematodes

The lineage leading to *C. elegans* lost DNMT1 and DNMT3 ∼350my ago [36]. However, some nematodes retain DNA methylation [36,70]. All nematodes with DNA methylation show some evidence of TE methylation, and no compelling evidence of methylation of genes [36]. Unusually, the nematode lineage leading to the mammalian parasites *Trichinella spiralis Trichuris muris* has retained DNMT3 but lost DNMT1, an unusual configuration across eukaryotes. In the nematode *T. spiralis* DNA methylation is associated with silencing by small RNAs [71] but it is not clear whether this is ancestral to the phylum. Loss of DNA methylation in nematode evolution correlates to the appearance of a new type of small RNA pathway, characterised by short RNA dependent RNA polymerase products known as 22G-RNAs [71], which silence TEs [72]; this may have enabled loss of DNA methylation by protecting the genome in its absence.

### DNA methylation in arthropods

The honey bee was the first animal species outside of mammals to be subjected to whole genome bisulfite sequencing, which led to a long-held perception that arthropods, and indeed invertebrates more widely had methylation at a subset of highly expressed genes, with no methylation at TEs [42]. The honey bee and other hymenoptera as well as coeloptera display very low levels of methylation and a small subset of genes are methylated [39,40,47]. However, further back in arthropod evolution there is evidence of TE methylation [43,45,48]. Ancestral state reconstruction revealed that the most likely state involved ∼10% methylation of CG sequences in TEs and slightly higher in genes [48].

Across arthropods methylation at a subset of orthologous genes seems to have been conserved across ∼300my of evolution [48]. The mechanism whereby this occurs is likely to be distinct from the H3K36me3-directed mechanism observed in mammals, because similar genes are methylated even in lineages that have lost DNMT3 and only have DNMT1. Methylated genes are enriched for housekeeping functions and, interestingly, display ‘focussed' expression [26,44,48,73]. By mapping these genes to their *Drosophila* orthologues, it was shown that they were likely to have broad rather than tissue-specific expression [48]. This correlated to a characteristic nucleosome arrangement around the transcription start site and a broad transcription initiation region, characteristic of housekeeping genes [74]. Since *Drosophila* orthologues have these characteristics despite complete absence of CG methylation, it is likely that methylation is a consequence of the distinct nucleosome environment rather than a cause [48]. This offers a possible mechanistic explanation for why certain genes acquire methylation in arthropods.

Although low level TE methylation is widespread across arthropods methylation of particular TEs is not strongly associated with reduced TE expression [48]. However, two species, *S. maritima* and *P. citri*, show extensive TE methylation, and this correlates with the evolution of promoter methylation that is anticorrelated to gene expression, reminiscent of mammals [48]. In *S. maritima*, this may also be associated with the evolution of a novel family of DNA repeats [46]. Further study of other arthropod species will undoubtedly reveal further novel patterns of DNA methylation.

### DNA methylation in lophotrochozoa

Our understanding of methylation patterns in lophotrochozoans is limited. In the mollusc *Crassostrea gigas*, there is methylation at genes but not TEs [75]. DNA methylation shows positive correlation with gene expression, although further analysis would be needed to test whether this is similar to arthropods, where ‘moderate' expression is enriched within methylated genes [75]. Integration of this data with analysis of nucleosome positioning in this species would also be interesting.

Within lophotrochozoa, the platyhelminth phylum is potentially interesting for further study. Parasitic flatworms are monophyletic and have lost DNMT1 and DNMT3 at the base of the group, alongside ALKB2 [76]. One report suggested that DNMT2 could methylate cytosine in DNA [20] but given findings from other organisms presented above, this may indicate false positive methylation or off-target, low-level methylation that is not relevant to its cellular role. The widely used free-living flatworm model organism *Schmidtea mediterranea* has lost DNMT1 and DNMT3, but some free-living flatworms, such as *Macrostomum ligano* [77], have retained both DNMT1 and DNMT3, alongside ALKB2. The patterns of methylation across the genome are unknown in any free-living flatworm.

### Methylation in early-branching animals

DNA methylation has been studied at nucleotide resolution in two sponges (*Amphimedon queenslandica* and *Sycon ciliatum*), a sea anemone (*Nematostella vectensis*) and a comb jelly (*Mnemiopsis leidyi*) [78]. Comparative analyses showed that CG methylation within genes was present in all these organisms, but that the overall levels of DNA methylation were highest in the sponge *A. queenslandica*, which displayed vertebrate levels of DNA methylation, methylation of TEs, and variable methylation of promoters suggesting a role for DNA methylation in regulating gene expression [78].

### Methylation patterns in plants

The evolution of methylation patterns in plants displays some similarities and some differences to that of animals. Extensive research has investigated methylation patterns in the model flowering plant *Arabidopsis* [13]. To summarise, CG methylation occurs at genes with moderate to high expression [10] and is associated with specific positions on the nucleosome [79], although it is not clear whether the relationship between positioned nucleosomes at the transcription start site and DNA methylation seen in arthropods is also present in *Arabidopsis*. Transposable elements and repeats have high levels of CG methylation and also are the sites of CHG and CHH methylation (where H = A or T), which has a silencing function [13]. Promoter methylation in the form seen in vertebrates is only rarely associated with silencing in *Arabidopsis*. However, sometimes protein-coding genes can be silenced by DNA methylation- this is due to acquisition of TE-like CHG and CHH methylation and can occur due to mutations where TE fragments jump into a protein-coding gene [80].

How well conserved are methylation patterns across plants? Within the Brassicaceae, close relatives of *Arabidopsis*, the overall patterns of methylation across the genome are largely conserved. Moreover, the level of methylation within the bodies of individual genes is conserved in orthologues in different species [81]. More widely, DNA methylation at gene bodies and TEs is generally conserved in flowering plants, although the levels of DNA methylation vary somewhat [82]. Nevertheless, the duckweed has lost gene body methylation and displays unusual TE methylation that is independent of small RNAs, suggesting that even within flowering plants, methylation patterns can evolve rapidly [83]. Interestingly, in contrast with conservation of gene body methylation in Brassicaceae, in maize, the specific genes that are subject to gene body methylation do not seem to be under strong purifying selection [84].

Outside of flowering plants, the distribution of methylation seen in *Arabidopsis* is not conserved. TE methylation is found in basal plants but the pattern of gene body methylation observed in *Arabidopsis* is not obvious, with the exception of the pine *P. tanae* and Charophytes such as *Klebsorbium nitens* [85–87]. Either recurrent gain of gene body methylation in some lineages including plants or loss of gene body methylation in many basal plant lineages are possible hypotheses to explain these patterns and further sampling is probably required to clarify this.

An important study on methylome evolution in plants identified two plant species *Eutrema salsugineum* and *Conringia planisiliqua* which have independently lost CG methylation at genes, correlating to the loss of the plant *de novo* methyltransferase CMT3 [88]. In these species the orthologues of genes with gene body methylation in closely related plants showed no difference in gene expression or gene expression variability [88,89] It was proposed that gene body methylation arises due to occasional random errors where CMT3 targets an expressed gene and introduces both CHG and CG methylation [87]. At transcribed genes, however, aberrant CHG methylation is lost because the transcription process recruits the H3K9 demethylase IMB1 [90]. In contrast, CG methylation can be maintained due to maintenance methyltransferases with little effect on transcription. In a direct test of this model, introduction of CMT3 into *E. salsugineum* causes acquisition of CG methylation that can be inherited in the absence of CMT3 [91].

Two key aspects required for this model are open to challenge. First, the model relies on faithful maintenance of methylation states through DNA replication but the stability of epimutations through long-term evolution experiments in *Arabidopsis* is limited [92–94]. It is possible, though, that epimutations spanning entire genes may have greater stability due to cooperativity between individual methylated cytosines [95]. Second, the apparent lack of effect on gene expression from DNA methylation has been challenged by comparative evolutionary analyses [96,97]. Laboratory experiments are much less sensitive to subtle fitness effects than evolution over millions of years. Nevertheless, this fascinating model has great potential to revise the assumption that notable features of genome-wide methylation patterns are adaptive and involved in gene regulation.

### Methylation patterns in fungi

Comparative analysis of DNA methylation across fungi was the subject of a recent extensive study of 40 genomes by whole genome bisulfite sequencing to map DNA methylation [50]. Across fungi there was no evidence of gene body methylation of CG sequences. Instead, the CG methylation was frequently found at repeats. Indeed, the proportion of the genome made up of repetitive elements was a significant predictor of the genome-wide level of CG methylation [50]. This strongly implies that silencing of repetitive elements is the ancestral role of TE methylation in fungi.

Within fungi, some interesting examples of DNA methylation in TE regulation have evolved. Repeat induced point mutation, with the memorable acronym RIP, was first characterised in *Neurospora crassa* [98] but is present in many other Ascomycota [99]. RIP involves the activity of the cytosine methyltransferase Masc1 (also known as RID), which is recruited to repetitive DNA during meiosis and is associated with an extremely high rate of C-T transitions [100]. The mechanism is still unclear, because the endogenous rate of deamination is probably too slow to account for the rapid rate of mutagenesis [101]. There may be a specific enzyme that mediates deamination of methylated cytosine. An alternative possibility is that Masc1 itself catalyses this reaction [100]. So far this is the only known exploitation of the mutagenic properties of cytosine methylation but it may well have evolved in other species outside fungi.

Another widespread type of methylation in fungi that is likely TE-specific is associated with DNMT5 [7]. DNMT5 is thought to have maintenance activity [7,50], which has been demonstrated biochemically for the *C. neoformans* enzyme [8]. The evidence for a TE-directed role of DNMT5 comes from analysis of *C. neoformans*, which only has DNMT5 and where methylation is restricted to a subset of repeats concentrated at centromeres [7,8]. In other species with DNMT5 TE methylation is also seen [39,50] but as DNMT1 is present it is not clear whether DNMT5 is also required for this modification.

### Other eukaryotic methylomes

The methylomes of the diverse group of single celled organisms at the base of the eukaryotic tree have so far been understudied. Many protists have lost DNA methylation completely, however, there are a number of organisms that retain DNA methylation [36] and a systematic study of these is still missing. One important study investigated methylation patterns in several brown and green algal species, finding genome-wide, highly periodic methylation linked to DNMT5 [7]. In the alga *Emiliania huxleyi* the periodicity was associated with nucleosome organisation, with methylation confined to linker regions of the DNA. This striking methylation pattern seems to have arisen specifically in this algal lineage [7]. Evidently, new patterns of DNA methylation to evolve even using the same machinery as found in other organisms and highlighting the need for more investigations in early-branching eukaryotes to clarify the ancestral state of the methylome.

### Evolutionary history of the eukaryotic methylome

Gathering together the studies across eukaryotes described above, three possible hypotheses to explain the distribution of methylation patterns can be proposed.

**Figure 1.**
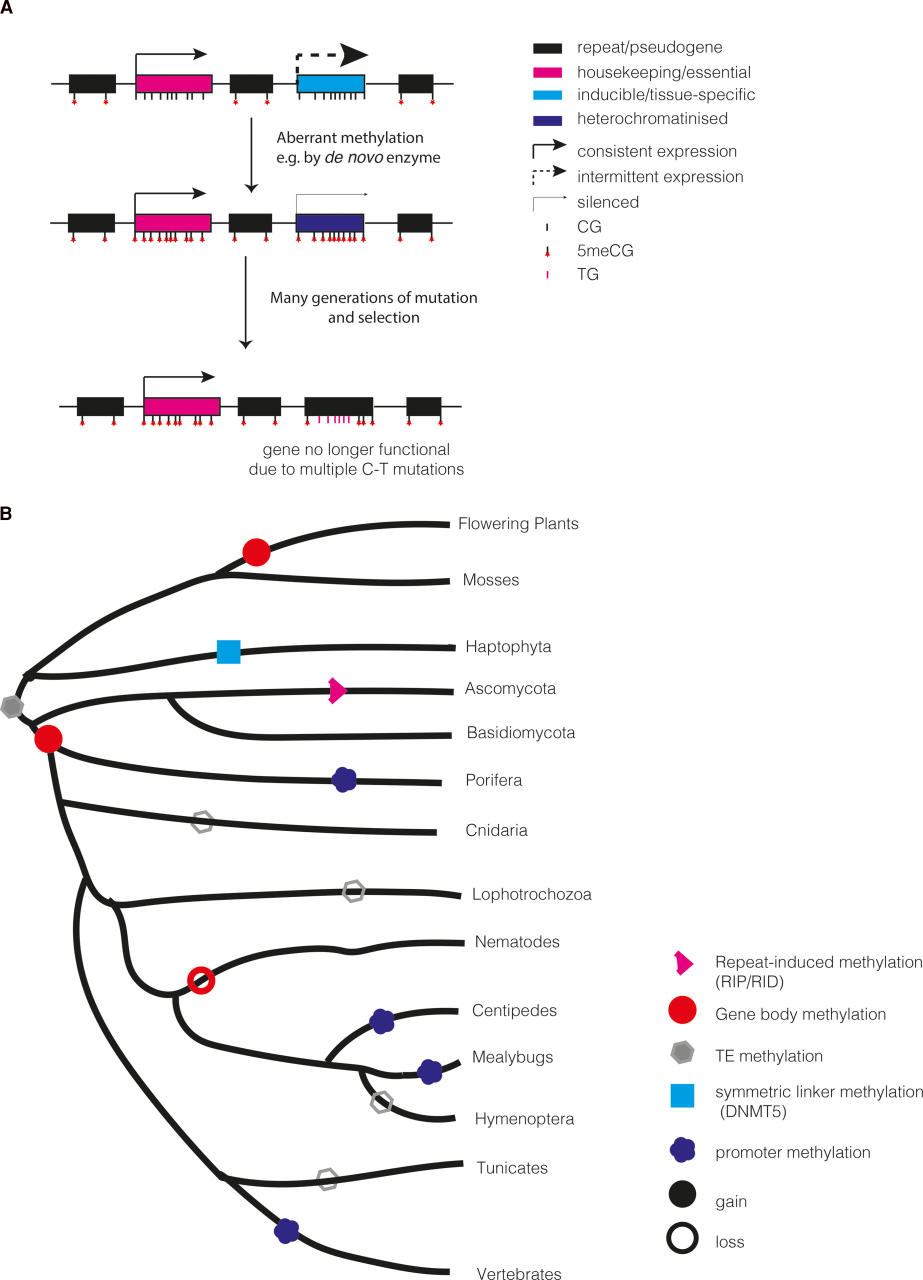
New hypotheses on the evolution of methylation across eukaryotes. (**A**) A model for how gene body methylation might be a consequence of evolutionary processes. Any gene could aberrantly acquire CG methylation, but in an inducible or tissue-specific gene, selection to maintain the gene is weaker so the increased mutation rate associated with 5mC leads to loss of gene function, whilst purifying selection preserves non housekeeping genes. (**B**) Recurrent acquisition of gene body methylation and loss of TE methylation characterises eukaryotic DNA methylation patterns. The guide tree is for illustration purposes only and branch lengths are not accurate. Only lineages in which some form of DNA methylation is retained are shown.

1) Gene body methylation of moderately expressed genes and dense TE methylation associated with TE silencing were ancestral, but either or both of these have been lost in individual lineages. This would be consistent with retention of both of these features in plants and animals. A slightly modified hypothesis to fit with the apparent rarity of TE methylation across animals would be that TE methylation was lost in primitive metazoa but regained in a few lineages such as vertebrates, sponge, nematodes and some arthropods [78].
2) Gene body methylation was ancestral to eukaryotes but TE methylation evolved independently in vertebrates, plants, and some fungal lineages. The main factor in favour of this model is the fact that many animal lineages display some form of gene body methylation, and that gene body methylation seems to be targeted to genes with similar characteristics in plants and many animal species [32].
3) Inspired by new thinking about gene body methylation in plants [87,91], TE methylation might have been ancestral to all eukaryotes but gene body methylation was not. Instead, gene body methylation arose repeatedly due to mistargeted TE methylation occasionally attacking genes (Figure 1A,B). In the original proposal for plants, this occurs due to CMT3 non-CG methylation [87], but other *de novo* methylation enzymes or even aberrant activity from a maintenance methyltransferase could initiate this process. Due to the ability of DNA methylation to be propagated through cell division, methylation of certain genes would persist. Two factors would make this more likely to happen at housekeeping genes:
  i)  Consistent expression of housekeeping genes might resist heterochromatin formation, as in plants [91]
  ii) Over long periods of time, selection to maintain sequence would be stronger at housekeeping genes than non-essential genes. As a result, C to T mutations that are promoted by methylation [27] would accumulate only in non-essential genes, causing them to degrade and eventually no longer be recognisable. Even if the origin of gene body methylation was independent in plants and animals, similar biases in the types of genes subject to methylation would emerge.

Hypothesis 3 underlies a very important point- that some of the features of methylomes recurring in different organisms could reflect constraints from the basic molecular biology of methyltransferases, which might not indicate conserved evolutionary history of the genome-wide methylation pattern. Another example of this may be the influence of nucleosome positioning on methylation patterns observed in plants, mammals and arthropods [48,79,102]. It also suggests that DNA methylation could be propagated independent of any functional benefit but that is coopted throughout evolution into different functional roles, a concept familiar from hypotheses concerning retrotransposons [103].

## Perspectives

- Methylomes often differ considerably even between closely related species and so examination of many phylogenetically distant species is vital to understand its evolution. New techniques to simultaneously acquire methylomes and genomes rapidly and cheaply should enable many more eukaryotic organisms to be characterised, which will assist ancestral state reconstruction to propose plausible hypotheses for key nodes on the eukaryotic family tree.
- Specific DNA methylation patterns may not always be the product of adaptive natural selection. New hypotheses about how DNA methylation could be maintained without any specific benefit to organisms have the potential to inspire new ways of thinking about DNA methylation evolution.
- Cancer methylomes are highly variable [104] and much of this is poorly understood [105]. Understanding some of the factors, including passive or selfish DNA methylation maintenance, that lead to changes in methylomes across species, could be applied to the rapidly evolving cancer cell and prompt new ideas for treatment.

## Abbreviations

RIP: Repeat-induced point mutation
TE: transposable element
